# The concept of Evil in Psychiatry: Philosophy, neurobiology and clinical implications

**DOI:** 10.1192/j.eurpsy.2022.1726

**Published:** 2022-09-01

**Authors:** A. Maia, S. Nascimento

**Affiliations:** 1 Nova Medical School, Mental Health, Lisbon, Portugal; 2 Centro Hospitalar de Lisboa Ocidental, Mental Health And Psychiatry, Lisbon, Portugal; 3 Champalimaud Centre for the Unknown, Neuropsychiatry Unit, Lisbon, Portugal; 4 Centro Hospitalar Psiquiátrico de Lisboa, General Psychiatry, Lisbon, Portugal

**Keywords:** Evil, Neurobiology, Antisocial Personality disorder, philosophy

## Abstract

**Introduction:**

Although difficult to define, the concept of evil is widely used and implicitly influences psychiatric judgements and diagnosis. Most definitions of evil rely on classical philosophical concepts, but it remains controversial if evil is a concept by itself or rather a dysfunction on the ability to experience goodness. Also, it is unclear if there is a neurobiological basis for evil or if it is entirely dependent on socio-cultural beliefs.

**Objectives:**

In this work, we intend to systematize evidence on the philosophical definitions and neurobiological correlates of evil, and reflect on its implications in clinical psychiatry.

**Methods:**

Literature review.

**Results:**

The concept of evil has been a theme of debate since the ancient Greek, where Plato argued that evil was a result of ignorance and Aristoteles saw morality as a guide for education and politics. Nietzsche claimed that evil was a dangerous concept that was created by the church, while Hannah Arendt underlined the banality of evil by highlighting “thoughtlessness” that frequently justify evil acts. From a neurobiological perspective, studies assessing individuals with neuro-psychiatric conditions associated with evil-related behavioral abnormalities have been suggesting a potential role of frontal and limbic structures, as well as of the serotonergic system. However, several of these studies assessed presumed correlates of evil, such as antisocial personality disorder or impulsive-aggressive behavior.

**Conclusions:**

Despite the polemic frontier between neurosciences and morality, a conceptual insight over the definition of evil is vital to guide comprehensiveness and clinical approach when dealing with deviant evil-like behaviors.

**Disclosure:**

No significant relationships.

